# Neuroendocrine apendicopathy in morphologically normal appendices of patients with diagnosis of acute appendicitis: Diagnostic study

**DOI:** 10.1016/j.amsu.2020.10.044

**Published:** 2020-11-06

**Authors:** Andy Petroianu, Thiago Vinicius Villar Barroso, Marcelo Araújo Buzelin, Bárbara De Melo Theobaldo, Luciene Simões De Assis Tafuri

**Affiliations:** Department of Surgery, School of Medicine, Federal University of Minas Gerais, Brazil

**Keywords:** Appendicitis, Diagnosis, Immunohistochemistry, Histology, Neuroendocrine peptide, Neuroimmune peptide, GIP, gastrin inhibitor peptide, Tryptase, mast cell-related tryptase, VEGFA, vascular endothelial growth factor, VIP, intestinal vasoactive peptide, TNFα, tumor necrosis factor alpha, IL-1, interleukin 1, PGE-2, prostaglandin E 2, PGP 9.5, gene-protein product 9.5, CD8, CD8 T lymphocytes, G1, group 1, G2, group 2, G3, group 3

## Abstract

**Background:**

About 15%–25% of appendices removed to treat acute appendicitis present normal macro- and macroscopic morphology. The objective of this study was to verify an association of proinflammatory, neuroendocrine and immune mediators with morphologically normal appendices removed from patients with clinical laboratorial and imaging characteristics of acute appendicitis.

**Materials and methods:**

Appendices removed from 121 adult patients of both genders were distributed into three groups according to their following characteristics: group 1: 53 macro- and microscopically normal appendices from patients with clinical, laboratorial and imaging diagnosis of acute appendicitis; group 2: 24 inflamed appendices from patients with clinical, laboratorial, imaging and histopathological diagnosis of acute appendicitis; group 3: 44 normal appendices from patients submitted to right colectomy to treat localized ascending colon adenocarcinoma. All appendices were immunohistochemically studied for gastrin inhibitor peptide, mast cell tryptase, vascular endothelial growth factor; intestinal vasoactive peptide, tumor necrosis factor alpha, interleukin 1, prostaglandin E2, gene-protein product 9.5, CD8 T lymphocytes, synaptophysine, enolase, and S100 protein.

**Results:**

The group 1 revealed increased levels of synaptophysine, enolase, mast cell tryptase and PGP-9.5 comparing with the other two groups. The group 2 presented increased levels of interleukin 1, CD8 T lymphocytes and prostaglandin E2 comparing with the other two groups. The group 3 confirmed the normal levels of all these neuroendocrine, immune and proinflammatory mediators.

**Conclusions:**

Morphologically normal appendices removed from patients with clinical and complementary exams indicating acute appendicitis have appendicular neuroimmunoendocrine disorder associated with the mediators synaptophysin, enolase, mast cell-related tryptase and gene-protein product 9.5.

## Highlights

• 15 % to 25% of the appendices removed to treat acute appendicitis have normal appearance with no inflammatory signs.
• All patients with normal appendices removed due to acute appendicitis do not present similar clinical manifestation anymore.
• Inflammatory and neuroendocrine appendicopathies present similar clinical laboratorial and imaging characteristics.
• Morphologically normal appendices removed from patients indicating acute appendicitis have neuroimmunoendocrine disorders.
• Synaptophysin, enolase, mast cell tryptase and gene-protein product 9.5 are associated with neuroimmunoendocrine disorders.

## 1 Introduction

Despite being the main cause of acute surgical abdomen, appendicitis has not been studied in the correct proportion of its relevance. The phylogenetic origin of the appendix, its functions and the pathophysiology of its diseases remain unknown [1,2]. The lack of understanding of the etiopathogenesis of most appendicular diseases makes appendectomy the standard treatment [3,4]. About 15% to 25% of the appendices removed to treat acute appendicitis diagnosed by clinical data, laboratory and imaging tests show no inflammatory signs and their macro- and microscopic appearance is normal [1,[5], [6], [7]]. Even so, immediately after appendectomy, all symptoms, signs and disorders in the complementary exams disappear permanently [1,[8], [9], [10]]. This situation raises doubts as to whether the removed appendix was really normal, as indicated by the histological study.

The etiopathogenesis of acute appendicitis is attributed to intraluminal obstructive factors, which result in distension of the organ and impaired blood circulation, resulting in the invasion of its wall by microorganisms [11,12]. However, this widely accepted theory has not clinically or experimentally been proved [7,13]. New evidences have shown caecal distention with fecal retention in a local ileum probably due to inflammatory and neuroendocrine effect [[14], [15], [16]].

Maresch and Masson (1921) called the inflamed appendiceal disease neurogenic appendicitis, because these authors considered the appendiceal histological aspect similar to that of inflamed neuromas [17,18]. Hofler et al. (1980) found in the inflamed appendices an increase in the number of neurofibers and proposed to change the term appendicitis by neurogenic appendicopathy [19]. On the other hand, Guller et al. (2001) considered neurogenic appendicopathy and acute appendicitis different diseases with a similar clinical picture [20]. More recently, neuroendocrine changes have been found in apparently normal appendices removed from patients with diagnosis of acute appendicitis [1,[21], [22], [23], [24], [25]].

The purpose of this study was to verify there is an association of proinflammatory, neuroendocrine and immune mediators with morphologically normal appendices removed from patients with clinical laboratorial and imaging characteristics of acute appendicitis.

## 2 Materials and methods

This work has been reported in line with the STARD (Standards for the Reporting of Diagnostic Accuracy Studies) criteria and it is part of a line of research on acute appendicitis approved by the Ethics Committee of the … … … ……, under protocol number 0429/06. [… … … …. ]

Appendices removed from 121 adult patients of both genders, were distributed into the following three groups according to their characteristics: group 1: 53 macro- and microscopically normal appendices from patients with clinical, laboratorial and imaging diagnosis of acute appendicitis, without any other disease; group 2: 24 inflamed appendices from patients with clinical, laboratorial, imaging and histopathological diagnosis of acute appendicitis, without any other disease; group 3: 44 normal appendices from patients submitted to right colectomy to treat localized ascending colon adenocarcinoma, without any other disease.

All patients in groups 1 and 2 were diagnosed with acute appendicitis based on the clinical picture of pain in the right flank, loss of appetite and nausea. Laboratory tests revealed leukocytosis with a predominance of polymorphonuclear cells. Acute appendicitis was confirmed by characteristic radiological, ultrasound and tomographic immages, including fecal loading in a distended cecum, appendiceal thickening and periapendicular fluid [3,24,26]. The appendices of group 3, considered as control, were all morphologically normal and no patient presented any complaint related to the appendix. The ascending colon tumor was located more than 10 cm far from the cecum in all cases.

The appendices of the three groups were processed by routine pathological examination with hematoxylin and eosin staining and analyzed under an optical microscope by two different pathologists without one knowing the other's report, nor the clinical history of the patients.

The immunohistochemical analysis was performed in 4 μm histological sections from the appendices included in paraffin, using the polymer method, with 3′,3′diamonobenzidine staining and polymer detection system HI DEF Detection, HRP Polymer System, Cell Marque brand. Table 1 shows the mediators that were studied in this work.

**Table 1:** **-** Antibodies used, characterized by clone, laboratory, dilution and pH.GIP: gastrin inhibitor peptide; Tryptase: mast cell-related tryptase; VEGFA: vascular endothelial growth factor; VIP: intestinal vasoactive peptide; TNFα: tumor necrosis factor alpha; IL-1: interleukin 1; PGE-2: prostaglandin E 2; PGP 9.5: gene-protein product 9.5; CD8: CD8 T lymphocytes.

Antibody	Clone	Laboratory	Dilution	pH
GIP	4	Enzo	1:75	6
Tryptase	AA1	DAKO	1:200	6
VEGFA	Policlonal	ABCAM	1:200	9
VIP	Policlonal	ABCAM	1:50	9
TNFα	2C8	FITZGERALD	1:100	6
Enolase	BBS/NC/VI-H14	DAKO	No dilution	9
IL-1	Policlonal	ABCAM	1:800	6
PGE-2	Policlonal	BIOSS	1:200	9
PGP 9.5	3D9	FITZGERALD	1:500	6
CD8	C8/144B	DAKO	No dilution	9
S-100 protein	4C4.9	DAKO	No dilution	9
Synaptophysin	DAK-SYNAP	DAKO	No dilution	6

The appendiceal sections included in paraffin were deparaffinized, fixed on glass slides and then diafinized by the method of inclusion, followed by xylol and alcohol battery, in the pathological routine. Then, the slides were dished in a buffer preheated to 95 °C to pH 6 or pH 9, according to the specification of each primary antibody for 1 h. Then, the reservoir containing the slides within the antigenic reactivation buffer were naturally cooled and dipped in TBST buffer at a pH 7.5 to 7.6 for 5 min. The slides were dried around the cuts to block endogenous peroxidase and immersed in a 3% hydrogen peroxide solution, then washed with TBST buffer for 5 min.

Each primary antibody was diluted according to its specifications and 100 μL were pipetted into each slide, which were incubated in a humid chamber for 60 min. The slides were washed with TBST buffer at a pH of 7.5–7.6, and 100 μL of the amplifying solution (DAKO Linker) was added over each slide to bind to several chains of secondary antibodies joined to the dextran polymer. By joining a primary antibody to several secondary antibodies, the chance of marker detection was increased. 100 μL of the detector solution (DAKO) formed by secondary antibodies joined by dextran polymer were pipetted. Secondary antibodies linked to dextran polymers promoted further unions with the 3′3′diaminobenzidine solution (Liquid DAB, Dako, USA. 100 μL of the 3′3′diaminobenzidine solution (Liquid DAB, Dako, USA). Appendiceal sections were counterstained with Harris' hematoxylin for 2 s and all slides were prepared using controls with other tissues known to be positive or negative for each marker, to avoid false positive or false negative results.

The slides submitted to immunohistochemical analysis were studied by a single pathologist without any knowledge about the group to which the appendiceal slide belong or any data related to patients. The immunomarked slide was observed in an optical microscope with a 400X definition. Each layer of the appendiceal wall (mucosa, submucosa, submucosa nerve plexus, muscle layer, myenteric nerve plexus and serosa) were studied in separate. The mediators’ antibodies expressions were characterized as follows:

- 0: immunostaining absent,
- 1: immunostaining present

The immunohistochemical staining percentages of all antibodies in the three groups were compared in each appendiceal layer as well as in the submucosal and myenteric nerve plexi separately. These studies were performed on percentages due to the discrepancy of the absolute values among the three groups. The results were compared using the chi-square test with Pearson's correction factor and Fisher's exact test, to determine the association of the immunostaining antibodies characteristics in the different constituents of the appendiceal wall in each group. The results were considered significant for a probability of significance greater than 95% (p < 0.05).

## 3 Results

There was no difference between the clinical and complementary diagnosis exams of groups 1 and 2. No difference was found in each group and among the three groups related to age and gender. On the first day after appendectomy, all clinical, laboratory, including leukocytosis, and imaging findings of all patients in groups 1 and 2 disappeared, and no longer occurred in the late postoperative follow-up.

There was no difference between the three groups in terms of gender distribution and the medians of age were group 1–22 (5–57) years; group 2–23 (10–45) years and group 3–64 (39–88) years, for being patients with cancer of the right colon.

**Fig. 1:**
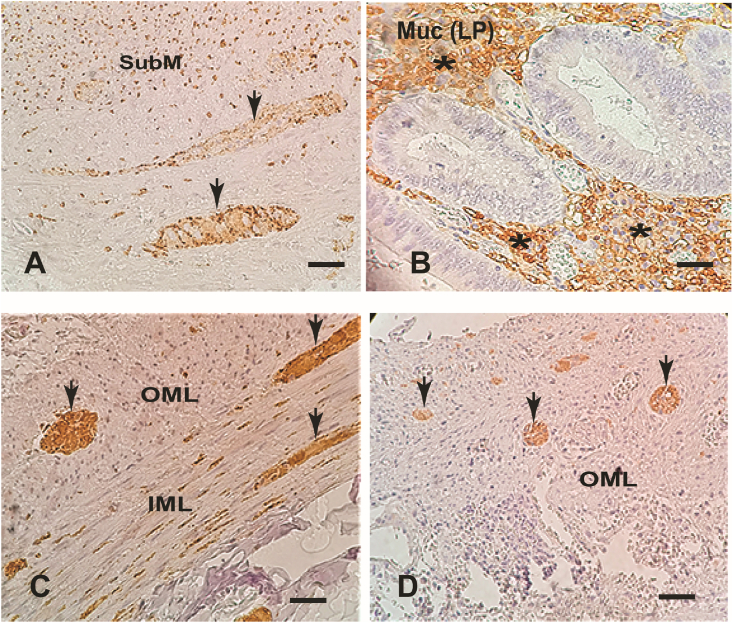
Microphotograph images showing positive mediators immunostaining (arrows) in the appendiceal wall (400 X):A - Synaptophysin in the submucosa nerve plexus and submucosa (SubM).B - Mast cell tryptase in the mucosa *lamina propria* (MucLP) (*).C - Enolase in myenteric nerve plexus, outer (OML) and inner (IML) muscle layer.D - PGP9.5 in the in myenteric nerve plexus.

When comparing the antibodies expressions in the appendiceal mucosa of the groups 1 and 2, there was greater expression for PGE2 and tryptase than in the normal mucosa of group 3. The expression of PGP-9.5 was much higher in Group 1 than in the other two groups, which practically did not express this protein (Table 2, Fig. 1D).

**Table 2:** **–** Comparison between the three studied groups regarding the percentage of positive immunohistochemical expression of all antibodies in the appendiceal mucosa.

	Grupo 1		Grupo 2		Grupo 3		
Mediadores	N	%	N	%	N	%	p
IL-1	53	51,0	24	54,2	44	28,6	0,069
PGE2	27	55,1	15	75,0	12	34,3	0,013* G1 = G2 > G3
CD8	37	80,4	18	78,3	21	80,8	1000
TNFα	25	55,6	5	20,8	12	42,9	**0,021** G1 > G2
Synaptophysin	33	73,3	12	50,0	28	53,6	0,093
Enolase	35	76,1	18	78,3	22	84,6	0,761*
S-100 protein	34	73,9	16	66,7	22	81,5	0,481
PGP-9.5	20	42,6	0	0,0	1	2,7	< 0,001* G1 > (G2 = G3)
Tryptase	43	89,6	12	52,2	12	32,4	**< 0,001** G1 > (G2 = G3)
VIP	38	79,2	18	81,8	20	83,3	0,942*
GIP	35	81,4	12	60,0	23	82,1	0,149*
VEGF	35	72,9	19	90,5	20	83,3	0,254*

N: Total appendices; %: Percentage of positive immunohistochemical expression for each antibody, indicating the presence of its corresponding mediator.

Group 1 (G1): macro- and microscopic normal appendices of patients with clinical and complementary diagnostic exams of acute appendicitis.

Group 2 (G2): inflamed appendices of patients with clinical and complementary diagnostic exams of acute appendicitis.

Group 3 (G3): normal appendices of patients with ascending colon adenocarcinoma submitted to right colectomy.

GIP: gastrin inhibitor peptide; Tryptase: mast cell-related tryptase, VEGF: growth factor of vascular endothelium; VIP: intestinal vasoactive peptide; TNFα: tumor necrosis factor alpha; IL-1: interleukin 1; PGE2:prostaglandin E2; PGP 9.5: gene-protein product 9.5; CD8: CD8 T lymphocytes.

p: significance by Pearson's chi-square test; *: significance by Fisher's exact test.

Regarding the percentage of immunohistochemical staining of all antibodies in the submucosa, PGE2 had greater expression in the group 2 than in the other two groups, which did not differentiate each other. The expression of tryptase was higher in group 1. The positivity of the VIP was greater in the appendices known to be normal than in the other two groups, which did not differ from each other (Table 3).

**TABLE 3:** **–** Comparison between the three studied groups regarding the percentage of positive immunohistochemical expression of all antibodies in the appendiceal submucosa.

	Grupo 1		Grupo 2		Grupo 3		
Mediadores	N	%	N	%	N	%	p
IL-1	53	4,1	24	16,7	44	5,7	0,168*
PGE2	5	10,2	8	40,0	8	22,9	0,020* G2 > (G1 = G3)
CD8	35	76,1	18	78,3	18	69,2	0,736
TNFα	13	28,9	3	12,5	4	14,3	0,200*
Synaptophysin	10	22,2	4	16,7	6	21,4	0,899*
Enolase	26	56,5	13	56,5	13	50,0	0,850
S-100 protein	33	71,7	12	50,0	15	55,6	0,150
PGP-9.5	2	4,3	0	0,0	0	0,0	0,693*
Tryptase	42	87,5	12	52,2	8	21,6	**< 0,001** G1 > G2 > G3
VIP	0	0,0	0	0,0	7	29,2	< 0,001* G3 > (G1 = G2)
GIP	27	62,8	11	55,0	10	35,7	0,080
VEGF	3	6,3	3	14,3	2	8,3	0,554*

N: Total appendices; %: Percentage of positive immunohistochemical expression for each antibody, indicating the presence of its corresponding mediator.

Group 1 (G1): macro- and microscopic normal appendices of patients with clinical and complementary diagnostic exams of acute appendicitis.

Group 2 (G2): inflamed appendices of patients with clinical and complementary diagnostic exams of acute appendicitis.

Group 3 (G3): normal appendices of patients with ascending colon adenocarcinoma submitted to right colectomy.

GIP: gastrin inhibitor peptide; Tryptase: mast cell-related tryptase, VEGF: growth factor of vascular endothelium; VIP: intestinal vasoactive peptide; TNFα: tumor necrosis factor alpha; IL-1: interleukin 1; PGE2:prostaglandin E2; PGP 9.5: gene-protein product 9.5; CD8: CD8 T lymphocytes.

p: significance by Pearson's chi-square test; *: significance by Fisher's exact test.

In the submucosal nerve plexus, CD8 had greater expression in the normal appendices (group 3) known than in those with an clinical appendicitis (groups 1 and 2). PGP-9.5 was more immunostained in group 1 than in the other two groups, which did not differ from each other (Table 4, Fig. 1D).

**TABLE 4:** **–** Comparison between the three studied groups regarding the percentage of positive immunohistochemical expression of all antibodies in the appendiceal submucosa nerve plexus.

	Grupo 1		Grupo 2		Grupo 3		
Mediadores	N	%	N	%	N	%	p
IL-1	53	0,0	24	0,0	44	0,0	–
PGE2	4	8,2	2	10,0	8	22,9	0,165*
CD8	0	0,0	1	4,3	4	15,4	0,012* G3 > G1
TNFα	–	–	–	–	–	–	–
Synaptophysin	12	26,7	4	16,7	12	42,9	0,105
Enolase	27	57,7	16	69,6	18	69,2	0,554
S-100 protein	34	73,9	12	50,0	21	77,8	0,062
PGP-9.5	33	70,2	3	13,0	3	8,1	< 0,001* G1 > (G2 = G3)
Tryptase	1	2,1	0	0,0	1	2,7	1000*
VIP	33	68,8	18	81,8	13	54,2	0,131
GIP	–	–	–	–	–	–	–
VEGF	30	62,5	18	85,7	13	54,2	0,069

N: Total appendices; %: Percentage of positive immunohistochemical expression for each antibody, indicating the presence of its corresponding mediator.

Group 1 (G1): macro- and microscopic normal appendices of patients with clinical and complementary diagnostic exams of acute appendicitis.

Group 2 (G2): inflamed appendices of patients with clinical and complementary diagnostic exams of acute appendicitis.

Group 3 (G3): normal appendices of patients with ascending colon adenocarcinoma submitted to right colectomy.

GIP: gastrin inhibitor peptide; Tryptase: mast cell-related tryptase, VEGF: growth factor of.vascular endothelium; VIP: intestinal vasoactive peptide; TNFα: tumor necrosis factor alpha; IL-1: interleukin 1; PGE2:prostaglandin E2; PGP 9.5: gene-protein product 9.5; CD8: CD8 T lymphocytes.

p: significance by Pearson's chi-square test; *: significance by Fisher's exact test.

Synaptophysin, enolase, PGP-9.5 protein and mast cell-related tryptase presented greater expression in group 1 than in the other two groups (Fig. 1). VEGF was more expressed in the groups 1 and 2 than in group 3. VIP was more immunostained in group 3. CD8 was more expressed in groups 2 and 3 than in group 1 (Table 5).

**TABLE 5:** **–** Comparison between the three studied groups regarding the percentage of positive immunohistochemical expression of all antibodies in the appendiceal muscle layer.

	Grupo 1		Grupo 2		Grupo 3		
Mediadores	N	%	N	%	N	%	p
IL-1	53	6,1	24	12,5	44	2,9	0,319*
PGE2	4	8,2	3	15,0	5	14,3	0,559*
CD8	4	8,7	9	39,1	7	26,9	0,008* - (G2 = G3) > G1
TNFα	–	–	–	–	–	–	–
Synaptophysin	22	48,9	4	16,7	6	21,4	**0,008 - G**1 > (G2 = G3)
Enolase	30	65,2	3	13,0	16	61,5	**< 0,001 - (**G1 = G3) > G2
S-100 protein	32	69,6	13	54,2	13	48,1	0,160
PGP-9.5	18	38,3	1	4,3	2	5,4	< 0,001* - G1 > (G2 = G3)
Tryptase	37	77,1	12	52,2	3	8,1	**< 0,001 - G**1 > G2 > G3
VIP	3	6,3	0	0,0	9	37,5	< 0,001* - G3 > (G1 = G2)
GIP	3	7,0	2	10,0	4	14,3	0,601*
VEGF	5	10,4	2	9,5	8	33,3	0,046* - (G1 = G2) > G3

N: Total appendices; %: Percentage of positive immunohistochemical expression for each antibody, indicating the presence of its corresponding mediator.

Group 1 (G1): macro- and microscopic normal appendices of patients with clinical and complementary diagnostic exams of acute appendicitis.

Group 2 (G2): inflamed appendices of patients with clinical and complementary diagnostic exams of acute appendicitis.

Group 3 (G3): normal appendices of patients with ascending colon adenocarcinoma submitted to right colectomy.

GIP: gastrin inhibitor peptide; Tryptase: mast cell-related tryptase, VEGF: growth factor of vascular endothelium; VIP: intestinal vasoactive peptide; TNFα: tumor necrosis factor alpha; IL-1: interleukin 1; PGE2:prostaglandin E2; PGP 9.5: gene-protein product 9.5; CD8: CD8 T lymphocytes.

p: significance by Pearson's chi-square test; *: significance by Fisher's exact test.

The IL1, PGP 9.5 and PGE2 expressions were greater in the myenteric nerve plexus of group 2 than in groups 1 and 3, which did not differ each other (Table 6, Fig. 1D).

**TABLE 6:** **–** Comparison between the three studied groups regarding the percentage of positive immunohistochemical expression of all antibodies in the appendiceal myenteric plexus.

	Grupo 1		Grupo 2		Grupo 3		
Mediadores	N	%	N	%	N	%	p
IL-1	12	24,5	11	45,8	5	14,3	**0,024** G2 > (G1 = G3)
PGE2	15	30,6	13	65,0	11	31,4	**0,018** G2 > (G1 = G3)
CD8	0	0,0	1	4,3	1	3,8	0,263*
TNFα	–	–	–	–	–	–	–
Synaptophysin	37	82,2	21	87,5	21	75,0	0,526
Enolase	36	78,3	21	91,3	19	73,1	0,253*
S-100 protein	37	80,4	21	87,5	20	74,1	0,481*
PGP-9.5	39	83,0	12	52,2	4	10,8	**< 0,001** G1 > G2 > G3
Tryptase	4	8,3	0	0,0	1	2,7	0,287*
VIP	41	85,4	19	86,4	16	66,7	0,154*
GIP	–	–	–	–	–	–	–
VEGF	40	83,3	18	85,7	16	66,7	0,212

N: Total appendices; %: Percentage of positive immunohistochemical expression for each antibody, indicating the presence of its corresponding mediator.

Group 1 (G1): macro- and microscopic normal appendices of patients with clinical and complementary diagnostic exams of acute appendicitis.

Group 2 (G2): inflamed appendices of patients with clinical and complementary diagnostic exams of acute appendicitis.

Group 3 (G3): normal appendices of patients with ascending colon adenocarcinoma submitted to right colectomy.

GIP: gastrin inhibitor peptide; Tryptase: mast cell-related tryptase, VEGF: growth factor of vascular endothelium; VIP: intestinal vasoactive peptide; TNFα: tumor necrosis factor alpha; IL-1: interleukin 1; PGE2:prostaglandin E2; PGP 9.5: gene-protein product 9.5; CD8: CD8 T lymphocytes.

p: significance by Pearson's chi-square test; *: significance by Fisher's exact test.

Both TNFα and CD8 were more immunostained in the appendiceal serosa of group 2 than in groups 1 and 3, which did not differ each other (Table 7).

**TABLE 7:** **–** Comparison between the three studied groups regarding the percentage of positive immunohistochemical expression of all antibodies in the appendiceal serosa.

	Group 1		Group 2		Group 3		
Mediators	N	%	N	%	N	%	p
IL-1	53	0,0	24	0,0	44	0,0	–
PGE2	1	2,0	1	5,0	3	8,6	0,339*
CD8	2	4,3	7	30,4	2	7,7	0,006* G2 > (G1 = G3)
TNFα	0	0,0	3	12,5	1	3,6	0,025* G2 > G1
Synaptophysin	1	2,2	0	0,0	0	0,0	1000
Enolase	0	0,0	1	4,3	0	0,0	0,242*
S-100 protein	3	6,5	6	25,0	3	11,1	0,098*
PGP-9.5	0	0,0	0	0,0	0	0,0	–
Tryptase	4	8,3	4	17,4	4	10,8	0,493*
VIP	0	0,0	0	0,0	0	0,0	–
GIP	–	–	–	–	–	–	–
VEGF	35	72,9	19	90,5	20	83,3	0,254*

N: Total appendices; %: Percentage of positive immunohistochemical expression for each antibody, indicating the presence of its corresponding mediator.

Group 1 (G1): macro- and microscopic normal appendices of patients with clinical and complementary diagnostic exams of acute appendicitis.

Group 2 (G2): inflamed appendices of patients with clinical and complementary diagnostic exams of acute appendicitis.

Group 3 (G3): normal appendices of patients with ascending colon adenocarcinoma submitted to right colectomy.

GIP: gastrin inhibitor peptide; Tryptase: mast cell-related tryptase, VEGF: growth factor of vascular endothelium; VIP: intestinal vasoactive peptide; TNFα: tumor necrosis factor alpha; IL-1: interleukin 1; PGE2:prostaglandin E2; PGP 9.5: gene-protein product 9.5; CD8: CD8 T lymphocytes.

p: significance by Pearson's chi-square test; *: significance by Fisher's exact test.

## 4 Discussion

This study was retrospectively performed because only after the surgical procedure and histological exams, the characteristics of the appendices of the three groups could be confirmed. The limited number of patients was due to the selection of patients, who had no other disease except the appendicopathy (groups 1 and 2) or right colon cancer (group 3).

The clinical picture and all the complementary exams performed in patients in group 1 indicated appendiceal disease, however the pathological analysis did not find any disorder in their wall. Although no local inflammation was found, the appendices were removed and all clinical and complementary manifestations disappeared immediately after the surgical procedure. Therefore, it is worth to assume that there was a non-inflammatory appendiceal disease, which was treated by appendectomy. This research verified the possibility that the disorder may be related to the neuroimmunoendocrine mediators located in the appendix [27–27].

TNFα is an immunoinflammatory mediator that acts on the influx of leukocytes, promoting their adhesion to the endothelium and migration through the vessels [28], and its secretion is stimulated by microbial products, immune complexes, foreign bodies, trauma and inflammatory stimuli resulting from endothelial injury, with leukocyte activation and systemic response of the acute phase [29,30]. In this study, TNFα showed greater expressions in appendices known to be inflamed, in the serous layer and in the mucous layer of histologically normal appendices of group 1. Therefore, there is an immunoinflammatory reaction in the appendiceal mucosa even in the absence of an acute inflammatory manifestation. The increased TNFα expression is associated with fever and loss of appetite, which are also found in acute appendicitis [26].

Il-1 is an immunoinflammatory mediator that acts on the recruitment, adhesion and migration of leukocytes in blood vessels, being produced by endothelial cells stimulated by the systemic response of the acute phase, but not in the appendices without inflammation (groups 1 and 3). Nemeth et al. (2001) and Wang et al. (1999) observed increased expression of Il-1 only in the mucosa and *lamina propria* in presence of acute appendicitis, indicating that Il-1 is related to an inflammatory disease [26].

PGE-2 is an immunoinflammatory mediator that causes vasodilation and increases the venous permeability of the microcirculation associated with edema [31]. This mediator is also related to the pathophysiology of pain. PGE2 acts on the posterior hypothalamus, inhibiting temperature control and facilitating the onset of fever. 32 This mediator was more expressed in the mucosa, submucosa and myenteric plexus of the inflamed appendices, indicating that it is a selective inflammatory mediator.

T cells group 8 (CD8) are part of the immune system that act as cytotoxic T lymphocytes (CTL), by destroying microorganisms. 33 The CD8 protein is a co-receptor in the activation of T cells and its name derives from the recognition of the antigen receptor ligand. T-CD8 cells destroy cells that express antigens in the cytoplasm and produce cytokines [34]. CD8 was increased only in the presence of appendiceal inflammation. Kooij et al. (2016) described increased CD8 in presence of inflamed appendices after starting antibiotic therapy and the number of circulating lymphocytes decrease [35]. In the absence of appendiceal inflammation (groups 1 and 3), the CD8, did not increase, probably because no antigen stimulus occurred.

VEGF is a homodimetric protein with neurovascular action as an angiogenic factor after hypoxia, trauma and in neoplasms, stabilizing the endothelium. After tissue aggression, this mediator stimulates the migration of endothelial cells, capillary proliferation and microcirculation vasodilation, increasing vascular permeability associated with angiogenesis and edema [36]. Its increased expression in the muscle layer groups 1 and 2 indicate that its manifestation is both immunoinflammatory and neuroendocrine.

The gastrin inhibitor polypeptide (GIP) is a neuroendocrine mediator secreted by K cells in the duodenum, jejunum, whose function is to increase the secretion of insulin and glucagon, in addition to inhibiting gastric hydrochloric acid excretion [37]. The expression of this mediator was greater in non-inflammatory appendicopathies, reinforcing the theory that this is a neuroendocrine disorder [38].

VIP is a neuroendocrine polypeptide present in the myenteric plexus and brain tissue. Its multiple functions include bronchodilation and gastrointestinal hydroelectrolytic excretion [7,24]. Di Sebastiano et al. (1999) and Bouchard et al. (2001) found its increase expression in the mucosa, submucosa and muscle layer of appendices without inflammation, removed by clinical picture of appendicitis, which these authors called neuroimmune appendicopathy. According to them, VIP is associated with appendiceal pain [7,24]. The studies by Barroso et al. (2015) found an increase in VIP expression also in inflamed appendices, not excluding the association of this mediator with pain [6].

Synaptophysin is a membrane glycoprotein, with a neuroendocrine mediating function associated with presynaptic vesicles expressed in neurons and diffuse cells of the neuroendocrine system (Fig. 1A). This mediator acts as a marker of neuronal and neuroendocrine neoplasms [39,40]. In this study, there was a high expression of synaptophysin in the muscle layer of group 1. Xiong et al. (2000) also found great expression of this mediator in morphologically normal appendices of patients with a clinical picture of acute appendicitis, reinforcing the idea of the real existence of neuroendocrine appendicopathy [25].

Enolase is a specific neuroendocrine mediator for neurons, acting as an isomer of the glycolytic enzyme enolase, identified in normal and neoplastic neuroendocrine cells (Fig. 1C). [41] Both in this work and in the studies by Xiong et al. (2000), there was greater expression of enolase in the appendiceal wall without inflammation in patients with clinical manifestation of acute appendicitis [25].

Mast cells belong to the immune inflammatory system derived from bone marrow and are activated by cross-linking affinity for immunoglobulin E (IgE) and anaphylatoxin receptors (Fig. 1B). Chemokines and physical stimuli cause mast cells to release immune-inflammatory mediators, such as leukotrienes, prostaglandins and cytokines stored in their granules [42]. Mast cells are microanatomically and functionally connected with peripheral nerves, resulting in a homeostatic unit in intestinal and defense neuroendocrine regulation, with the release of mediators that cause nausea, vomiting, abdominal pain and diarrhea, characteristic of the acute appendicitis [43]. This study found high expression of mast cell-related tryptase in the mucosa, submucosa, and serosa layers of non-inflamed appendices removed from patients with a clinical picture of acute appendicitis (group1). Manga et al. (2016) and Bhramaramba et al. (2016) also showed an increase in mast cells and neuronal hypertrophy in non-inflamed appendices of patients with an acute appendicitis clinic [44]. This association of neuroendocrine mediators with mediators of the systemic defense system indicate that this appendicopathy may actually be neuroimmunoendocrine.

PGP-9.5 is a neuroendocrine mediator belonging to the ubiquitin hydrolase protein family, isolated from the brain whose antibodies are used as markers of neurons and neuronal and neuroendocrine differentiating cells (Fig. 1D). [45] In this study, there was high expression of PGP-9.5 in the mucosa, submucosa, muscle layer and myenteric nerve plexus of non-inflamed appendices taken from patients with a clinical picture of acute appendicitis (group 1). Since the beginning of the 20th century, PGP-9.5 has been associated with an increase in the density of nerve fibers in neurogenic appendicopathy without signs of acute inflammation (Fig. 1D). [1,7,17–20,23-26.46,47].

The S-100 protein is a neuronal mediator characterized as a calcium-binding protein and expressed by neurons associated with pain [48]. In this study, there was almost a greater expression of this protein in the serosa of inflamed appendices. Partecke et al. (2013), Manga et al. (2016) and Ruiz et al. (2017) found an increase in S-100 in nerve fibers from morphologically normal appendices [18,44,46,47].

Immunoinflammatory markers (Il-1, PGE2, TNFα, CD8) had greater expression only in the inflamed appendices. All neuroimmunoendocrine mediators (synaptophysine, enolase, mast cell tryptase, PGP-9.5 and protein S100) had greater expression only in group 1 of morphologically normal appendices of patients with clinical manifestations of acute appendicitis and were probably responsible for nausea, vomiting, abdominal pain and diarrhea (Fig. 1). [66,70] Fever can be mediated by TNFα and Il-1 and pain by tryptase and PGE2. ^26,32^ Cecum stool stasis due to local adynamic ileus is another neuroimmunoendocrine manifestation that reinforces this appendicopathy [3,14,16,49,50].

The increased expressions of neuroendocrine and immune mediators indicate that appendicopathy currently called neurocrine, neuroendocrine and neruoimmunoendocrine is real. This disease occurs at an age and sex similar to that of acute appendicitis and manifests itself with clinical and complementary diagnosis exams similar to that of the inflamed appendix. However, its etiology, its activating factors and its pathophysiology are still unknown. Until the natural evolution of this disease will be known, appendectomy remains the standard treatment. Neuroimmunoendocrine appendicopathy is not an inflammatory disease and apparently is not related to infection, therefore the conservative treatment with antibiotics, proposed for acute appendicitis, has no scientific basis to be indicated.

According to our previous literature review of all articles related to non appendicopathies with symptoms of acute appendicitis, this is the largest study of mediators present in normal appendices, acute appendicitis and neuroimmunoendocrine appendicopathies [1]. This study demonstrated the mediators directly associated with inflamed and those with non-inflamed appendicopathies. This is in fact a novelty and show a new knowledge not previously published, which may be an advance in appendicitis research front, modifying the concept of normal appendices of patients with clinical and complementary exams indicating acute appendicitis.

## 5 Cconclusion

Morphologically normal appendices removed from patients with clinical and complementary exams indicating acute appendicitis have appendiceal neuroimmunoendocrine disorder associated with the mediators synaptophysin, enolase, mast cell-related tryptase and gene-protein product 9.5.

## Annals of medicine and surgery author disclosure form

The following additional information is required for submission. Please note that failure to respond to these questions/statements will mean your submission will be returned. If you have nothing to declare in any of these categories, then this should be stated.

Please state any conflicts of interest.

The authors, Andy Petroianu, Thiago Vinicius Villar Barroso, Marcelo Araújo Buzelin, Bárbara de Melo Theobaldo, Luciene Simões de Assis Tafuri declare no conflict of interest related to the work and manuscript entitled “Neuroendocrine apendicopathy in morphologically normal appendices of patients with diagnosis of acute appendicitis: Diagnostic study".

Please state any sources of funding for your research This Work and Article have no source of funding to support it. Please state whether Ethical Approval was given, by whom and the relevant Judgement's reference number This study is part of a line of research on acute appendicitis approved by the Ethics Committee of the Federal University of Minas Gerais, under protocol number 0429/06.

## Research registration unique identifying number (UIN)

Please enter the name of the registry, the hyperlink to the registration and the unique identifying number of the study. You can register your research at http://www.researchregistry.com to obtain your UIN if you have not already registered your study. This is mandatory for human studies only.

1. Name of the registry: Neuroendocrine apendicopathy in morphologically normal appendices of patients with diagnosis of acute appendicitis: Diagnostic study.

2. Unique Identifying number or registration ID: research registry 5936

3. Hyperlink to your specific registration (must be publicly accessible and will be checked): https://www.researchregistry.com/browse-the-registry#home/?view_2_search=research%20registry%205936&view_2_page=1.

## Guarantor

The Guarantor is the one or more people who accept full responsibility for the work and/or the conduct of the study, had access to the data, and controlled the decision to publish. Please note that providing a guarantor is compulsory.

Andy Petroianu is the Guarantor for this Work and Article.

Author contribution

Conception and design the study, analysis and interpretation of data, was the surgeon of several patients included in this study, participate in drafting the article and revising it critically and took responsibility for all aspects of this work and article.

Acquisition of data, analysis and interpretation of data, and participate in drafting the article.

Performed the immunohistological study, analysis and interpretation of data, participated in discussions.

Acquisition of data, analysis and interpretation of data, participated in discussions.

Performed the immunohistological study, analysis and interpretation of data, participate in drafting the article and revising it critically.

All authors accept direct responsibility for the manuscript and gave final approval of the version to be published.

## Provenance and peer review

Not commissioned, externally peer reviewed.

## Declaration of competing interest

The authors declare no conflict or competing interest with respect to the research, authorship and publication of this article. The authors have no financial relationship with any organization. The authors have full control of all data, and agree to allow the journal to review any data if requested.
