# Supplementary material for: Neuroendocrine apendicopathy in morphologically normal appendices of patients with diagnosis of acute appendicitis: Diagnostic study
Source: Ann Med Surg (Lond). 2020 Nov 6;60:344–51. doi: 10.1016/j.amsu.2020.10.044 (PMC7666303; doi:10.1016/j.amsu.2020.10.044)
Supplement: Multimedia component 1 [file mmc1.docx]

**NEUROENDOCRINE APENDICOPATHY IN MORPHOLOGICALLY NORMAL APPENDICES OF PATIENTS WITH DIAGNOSIS OF ACUTE APPENDICITIS: DIAGNOSTIC STUDY**

**STARD FLOW DIAGRAM**

**121 eligible participants, adults, both sexes with similar ages**

Group 1 (n = 53) Group 2 (n = 24) Group 3 (n = 44)

Clinics of appendicitis Clinics of appendicitis Right colon cancer

Normal appendices Acute appendicitis Normal appendices

**Immunohistochemical studies on all appendices**

Gastrin inhibitor peptide Mast cell tryptase Vascular endothelial growth factor

Intestinal vasoactive peptide Tumor necrosis factor alpha Interleukin 1

Prostaglandin E2 Gene-protein product 9.5 CD8 T lymphocytes

Synaptophysine Enolase S100 protein

**Clinics indicating acute appendicitis with morphologically normal appendices**

**Disorders**

Synaptophysin Enolase Mast cell-related tryptase Gene-protein product 9.5
