# Supplementary material for: Neuroendocrine apendicopathy in morphologically normal appendices of patients with diagnosis of acute appendicitis: Diagnostic study
Source: Ann Med Surg (Lond). 2020 Nov 6;60:344–51. doi: 10.1016/j.amsu.2020.10.044 (PMC7666303; doi:10.1016/j.amsu.2020.10.044)
Supplement: Multimedia component 2 [file mmc2.docx]

**NEUROENDOCRINE APENDICOPATHY IN MORPHOLOGICALLY NORMAL APPENDICES OF PATIENTS WITH DIAGNOSIS OF ACUTE APPENDICITIS: DIAGNOSTIC STUDY**

**DATA STATEMENT**

All data of this work can be immediately sent to the International Journal of Surgery if requested

**Results:** The group 1 revealed increased levels of synaptophysine, enolase, mast cell tryptase and PGP-9.5 comparing with the other two groups. The group 2 presented increased levels of interleukin 1, CD8 T lymphocytes and prostaglandin E2 comparing with the other two groups. The group 3 confirmed the normal levels of all these neuroendocrine, immune and proinflammatory mediators.

**Conclusions:** Morphologically normal appendices removed from patients with clinical and complementary exams indicating acute appendicitis have appendicular neuroimmunoendocrine disorder associated with the mediators synaptophysin, enolase, mast cell-related tryptase and gene-protein product 9.5.
